# Intermediate cell states in epithelial-to-mesenchymal transition

**DOI:** 10.1088/1478-3975/aaf928

**Published:** 2019-01-18

**Authors:** Yutong Sha, Daniel Haensel, Guadalupe Gutierrez, Huijing Du, Xing Dai, Qing Nie

**Affiliations:** 1 Department of Mathematics, University of California, Irvine, CA 92697, United States of America; 2 Department of Biological Chemistry, School of Medicine, University of California, Irvine, CA 92697, United States of America; 3 Department of Mathematics, University of Nebraska-Lincoln, Lincoln, NE 68588, United States of America; 4 Department of Development and Cell Biology, University of California, Irvine, CA 92697, United States of America; 5 Co-first authors.

**Keywords:** EMT, metastasis, stemness

## Abstract

The transition of epithelial cells into a mesenchymal state (epithelial-to-mesenchymal transition or EMT) is a highly dynamic process implicated in various biological processes. During EMT, cells do not necessarily exist in ‘pure’ epithelial or mesenchymal states. There are cells with mixed (or hybrid) features of the two, which are termed as the intermediate cell states (ICSs). While the exact functions of ICS remain elusive, together with EMT it appears to play important roles in embryogenesis, tissue development, and pathological processes such as cancer metastasis. Recent single cell experiments and advanced mathematical modeling have improved our capability in identifying ICS and provided a better understanding of ICS in development and disease. Here, we review the recent findings related to the ICS in/or EMT and highlight the challenges in the identification and functional characterization of ICS.

## 1. Introduction

The transition from epithelial to mesenchymal cells, as well as the reverse (mesenchymal-to-epithelial transition or MET), are highly dynamic processes implicated in various biological processes. EMT is well studied in the context of embryogenesis, organ fibrosis, and cancer metastasis, which highlight the categorical subtypes of EMT [ 1]. As our understanding of how EMT is regulated expands, it has become clear that EMT exists on a spectrum or continuum with various possible cell states existing between epithelial and mesenchymal phenotypes [2]. Cells do not necessarily exist in ‘pure’ epithelial or mesenchymal states, but instead can be in intermediate cell states (ICS) or hybrid states that possess characteristics of both epithelial and mesenchymal cells [2]. Context and tissue specific functions of EMT exist, but a number of key features and phenotypes are shared amongst the different subtypes of EMT [1, 2].

EMT involves the transient reduction and sometimes full loss of adhesion between epithelial cells. Hallmark changes include alterations in cytoskeleton architecture, changes in cell–cell and cell-matrix adhesions, and loss of apical-basal polarities [3]. In the past, EMT identification had been focused on the expression of a few markers such as E-cadherin (E-cad); however, as our understanding of EMT has progressed, an expanded view of EMT identification encompasses multiple layers of molecular changes including EMT transcription factors (EMT-TFs) and their gene regulatory networks that govern the transition [2]. A number of key EMT-TFs such as Snail1/Slug and Zeb1/2 have been identified in promoting the mesenchymal phenotype while other TFs such as Ovol1/2 and Grhl2 have been shown to suppress the mesenchymal phenotype thus promoting the epithelial phenotype [2, 3]. EMT regulation also occurs at the microRNA, long noncoding RNA, chromatin, and post-translational levels [2].

## 2. EMT in normal and diseased epithelial tissues

The development and maintenance of epithelial tissues is largely driven by epithelial stem cells [4]. The dynamic activities of these stem cells such as proliferation and differentiation vary depending on the stage of the tissue such as during development, adult regeneration, or in pathological conditions such as cancer [4]. Increasing evidence implicates EMT (and its regulation) as being another cellular dynamic component that can promote stem cell function [2]. In general, stem cell function in adult epithelial tissues is largely focused on the homeostatic maintenance of the tissue where cells that are differentiated or lost need to be replaced [4]. In line with the notion that EMT exists in a spectrum of states and degrees, its role can vary depending on the epithelial tissue, stage of development, whether the tissue is undergoing regeneration or repair, and the pathological condition [2]. Below we highlight several instances of EMT function and regulation in committed epithelial tissues in different contexts such as normal development, adult regeneration and repair, and briefly discuss in the context of cancer (readers are referred to other comprehensive reviews on this topic, e.g. [2]).

The normal and cancerous mammary epithelium has been utilized as a model to study the potential role and regulation of EMT in epithelial stem cell function. Experiments using immortalized human mammary epithelial cells provided *in vitro* evidence that ectopic expression of EMT-TFs Snail1 and Twist leads to acquisition of stem-like activities [5]. A positive correlation between heightened EMT gene signature and stem cell fate has also been noted for normal and cancerous mammary epithelial cell types in culture or directly isolated from the human or mouse tissue [6, 7]. These data generated much interest in EMT as they suggest a link between EMT and the gain of stem-like features.

Further work in the mammary model *in vivo* found that Snai2 (Slug) is the major EMT-TF expressed in mouse mammary basal cells known to contain multipotent stem cells, and that ectopic expression of Snai2 leads to enhanced stem-like features [8]. Moreover, knockout or knockdown of Snai2 compromises mammary epithelial development and/or the ability of primary mammary epithelial cells to regenerate a mammary tree [8, 9]. Zebl has also been found to be expressed in normal mouse and human mammary basal cells [10], with expression particularly enriched in the Procr^+^ stem cell subset [7]. However, its functional significance remains to be elucidated. Interestingly, using a transgenic mammary tumor model, Snail1-but not Snai2-expressing cells appeared in the early hyperplastic lesions as well as more high-grade carcinomas [11]. These cells lack E-cad expression and begin to express other EMT-TFs such as Zeb1, suggesting that Snail1 (but not Snai2) is responsible for governing the EMT program in cancer progression [11]. These observations highlight the notion that different EMT-TFs can have different, context-specific functions even in the same tissue, and the exact underlying molecular and cellular mechanisms may differ. Thus, we emphasize again the importance to now expand our view of EMT beyond a simple binary, linear or universally identical process with the end goal of generating mesenchymal cells. EMT can be thought of as a historical term that is redirected to describe the diverse and complex variant forms associated with epithelial-mesenchymal plasticity. Specifically, EMT may be considered a navigation through a rugged, highly nonlinear multidimensional landscape of different axes that cumulatively define EMT [12, 13]. On this landscape, cell states other than epithelial and mesenchymal cells often exist, exhibiting mixed (or hybrid) features of epithelial and mesenchymal states. Such cell states, termed as intermediate cell states (ICSs) in this paper, may play important roles in regulating transitions between epithelial cells and mesenchymal cells.

Growing evidence also points to the importance of regulating EMT during physiological epithelial development and regeneration. Within the mammary epithelium, suppression of EMT by Elf5 and Ovol2 TFs appears to be an integral component of its normal development and regeneration [14, 15]. Loss of Ovol2 in the mammary epithelium results in an up-regulation of a large number of EMT/mesenchymal markers such as vimentin (Vim) and EMT-TFs such as Zeb1, as well as *in vivo* morphological transformation reminiscent of EMT [15]. Importantly, many of these EMT genes are direct targets of Ovol2’s transcriptional repressor activity and depletion of Zeb1 rescues the regenerative defect caused by Ovol2 deficiency [15], underscoring an EMT-centric function of Ovol2 in the mammary gland. Interestingly and adding to the clinical importance of EMT regulation, incidence of metastasis-free survival increases in breast cancer patients with high levels of Ovol2 [15].

Transcriptional inhibition of EMT by Ovol2 and its homolog, Ovol1, is also critically important for normal skin epithelial development during embryogenesis. Loss of both Ovol2 and Ovol1 leads to defective epidermal and hair follicle morphogenesis [16]. Similar to the observations in the mammary gland, loss of Ovol leads to up-regulated expression of EMT structural markers and EMT-TFs, as well as EMT-like phenotypes such as reduced adhesion between, and aberrant migration of, embryonic epidermal cells [16]. In adult skin, loss of Ovol2 alone results in defective wound healing [17], a process that has been proposed to involve partial EMT of wound peripheral epidermal cells so they can efficiently migrate to close the wound [2, 18, 19]. Ovol2-deficient epidermal and hair follicle stem cells migrate faster than their normal counterparts, but with significantly reduced directionality [17]—defects that are near-completely rescued when EMT-TF Zeb1 is simultaneously lost. On the other hand, loss of EMT-TF Snai2 compromises epidermal migration after wounding, and results in a thin epidermis and transient delay of hair growth during normal development [ 19–21 ]. Together these data suggest that a delicate balance of EMT regulation exists to maintain both epithelial-like and mesenchymal-like states and this ability to toggle between the two states might be critical for skin epithelial development and regeneration.

The study of EMT in cancer has largely been focused on its role in promoting invasion and metastasis with an involvement in chemoresistance recently demonstrated [2]; however many questions remain unanswered. Conflicting literature exists and the extent of EMT importance in patients’ clinical outcomes remains unclear [2]. The roles of partial EMT and intermediate states in the context of cancer is still not well understood likely due to the transient nature of these events [2, 22]. The variation of different EMT-TFs’ expression together with the diverse cell types in the tissues of origin adds to the complexity. Below we delve deeper into the idea of intermediate states and discuss relevant existing data in the contexts of both normal and diseased cells.

## 3. Existence of ICS in EMT

During EMT, cells exist on a reversible spectrum, and can toggle between ICSs, which lie between epithelial and mesenchymal states. Several lines of evidence using *in vitro, in vivo,* and computational modeling suggest the existence of ICS.

*In vitro* models that have focused on using various cell lines are largely focused on the dual expression of epithelial and mesenchymal markers for identification of ICS. Glomerular parietal epithelial cells (GPECs) from adult mouse kidney adopt an intermediate phenotype expressing both epithelial and mesenchymal markers [23]. The MCF10A mammary epithelial cell line exists in an ICS with expression of both epithelial (E-cad) and mesenchymal (VIM) markers as compared to other well characterized breast cancer cell lines [24]. Cancerous cell lines have also been shown to exhibit ICS. Some cells from the human non-small-cell lung cancer cell line A549 are in an intermediate EMT state in culture based on co-expression of VIM and SNAI2 [25]. ITGB4^+^ triple-negative breast cancer stem cell-enriched cells reside in an intermediate state between ‘pure’ epithelial and mesenchymal cells [26]. Research from a number of groups indicates that individual carcinoma cells, rather than all cells at a population level, can exist in ICSs. Distinguished by the widely used cell-surface markers resolving epithelial and mesenchymal cells, single CD24^+^/CD44^+^ cells in tumorigenic human mammary epithelial cells are ICS cells [27]. H1975 lung cancer cells co-stained for both VIM and E-cad display an ICS phenotype at a single-cell level [28]. Circulating tumor cells from human breast cancer cell lines captured by a microfluidic device from blood exhibit both epithelial (MCF7 and SKBR3) and mesenchymal (MDA-MB-231) characteristics, indicating existence of potential ICSs [29]. The classical view of the metastatic cascade depicts a sequence of events including dissemination, invasion, intravasation, and eventually extravasation, and ICS cells expressing both epithelial and mesenchymal features can be observed at these different stages; however, dissecting the exact role that EMT plays at these stages *in vivo* has proven to be difficult [2].

Findings generated by modeling and computational approaches also support the idea of ICS. Modeling of the microRNA (miR)-based chimeric modules showed the miR-200/Zeb module functions as a ternary switch, allowing an intermediate phenotype in addition to the epithelial and mesenchymal phenotypes [30]. Random circuit perturbation (RACIPE), interrogating the robust dynamical behavior of a gene regulatory network, has identified two different types of intermediate phenotypes when applying to a proposed 22-gene network of EMT [31]. A topographic map using the combination of numerical simulations of a Boolean network model and the analysis of bulk and single-cell gene expression data revealed multiple ICSs, separating stable epithelial and mesenchymal states [32]. Energy-landscape based methods like scEpath could also be used to identify ICS [33] and the landscape of the free energy changes during the EMT of lung cancer cells suggests a stable ICS [34].

Previous studies predicted that multiple ICSs may exist between epithelial and mesenchymal states [35], but the number of intermediate phenotypes is still a matter of debate [36]. Recent experiments have provided evidence for one, two and three intermediate phenotypes from TGF-*β*-induced EMT in MCF10A cells [37], ovarian carcinoma (OC) cell lines [38], and circulating tumor cells in blood [29], respectively. While theoretical analysis helps to predict the number of ICSs, modeling studies have revealed that complex EMT regulatory networks govern the existence of multiple ICSs [39]. Modeling of miRNA-based regulation composed of a tristable circuit miR-200/Zeb driven by the monostable module miR-34/Snail1 allowed one intermediate EMT phenotype [30]. Ovol can modulate cellular plasticity by restricting EMT, driving MET, expanding the existence of the ICS and turning both EMT and MET into two-step processes based on the mechanism-based mathematical model coupling Ovol with the core regulatory network miR-200/Zeb [40]. Later, two distinct intermediate phenotypes in EMT dynamics were predicted and experimentally validated, and were shown to be modulated by Ovol2 and the Ovol2-Zeb1 mutual inhibition circuit [24]. Recently, analysis of a mathematical model that integrates expression data with the reported Tcf21-Slug interactions reveals one stable and two metastable ICSs in EMT [41].

## 4. Roles of ICS

### 4.1. Stemness

Recent theoretical and experimental studies have suggested that cells in ICSs can be more stem-like than both ‘pure’ epithelial and mesenchymal cells. For example, trophoblast stem cells isolated from the conceptuses of MAP3K4 kinase-inactive mouse (TS^KI4^) cells are found to be trapped in an ICS and exhibit properties of both EMT and sternness [42]. Other cells at ICS states may also acquire sternness. For example, co-culture of cells from E and M states actually enhances mammosphere formation (an assay for sternness), and the isolation of the CD24^+^/CD44^+^ hybrid E/M cell state leads to enhanced sternness [27]. In another example, more than 90% of CD24^+^ cells from kidney GPECs show co-expression of surface markers of renal progenitors CD24 and cadherin-11, suggesting that these cells have acquired stem cell-like properties [23].

Mathematical modeling and computational analysis also predict that the ICS confers stemness potential. A model on three-way switch LIN28/let-7 circuit and a model on EMT-STEM-Notch coupled circuit both revealed a high likelihood of an ICS phenotype (when compared with either epithelial or mesenchymal states) in gaining stemness [43, 44]. Moreover, the ICS location in the low-dimensional gene expression space can shift towards either end of the EMT spectrum, and a crosstalk between EMT and stem cell regulatory modules in conjunction with Notch signaling creates a window of opportunity for stemness [44]. If one could reduce the size of the window or shift the window away from the mesenchymal end of the EMT space, the tumor would be less aggressive in growth or invasiveness [45].

ICS importance has also been analyzed in other systems not directly related to EMT [39]. For example, based on a five-node stochastic gene regulatory network controlling cellular stemness, ICS is found to significantly decrease the barrier of potential landscape and the minimal action value along the transition path to promote the differentiation process [46]. Clearly, additional experimental studies are needed to clarify the mechanistic link between ICS and stemness, the relationship of which may be correlative rather than casual in some cases.

### 4.2. Collective migration

The ability of cells in ICS to migrate in a collective manner has been implicated during embryonic development, wound healing and the formation of tumor cell clusters [19, 28, 47, 48]. Cells undergoing collective migration display some migratory characteristics reminiscent of mesenchymal cells while maintain epithelial characteristics, implicating a connection to the ICS. For example, collective migration of H1975 lung cancer cells *in vitro* is associated with an ICS phenotype, and both are impaired after depletion of EMT-inhibiting TFs [28]. Cross-talks between Tcf21 and Slug have been identified to mediate phenotypic and migration plasticity in high-grade serous ovarian adenocarcinoma, and ICSs were found to be important in collective cell migration [41]. As discussed above, our recent studies highlight the importance of balancing the functions of EMT-inhibiting TF Ovol2 and EMT-TF Zeb1 in achieving directional cell migration of skin epithelial cells to support tissue repair and regeneration [17].

### 4.3. Drug resistance

The ICSs in EMT have great clinical relevance as they have been associated with drug resistance. The triplenegative breast cancer cells, which contain a number of intermediate E/M cells in primary tumor [29], exhibit *de novo* resistance to current standard therapies such as anthracyclines and taxanes [49, 50]. However, the underlying signaling pathways and molecular mechanisms of the interplay between ICS and drug resistance remain largely elusive [50].

### 4.4. Metastasis

The overarching role of EMT in metastasis has been extensively studied, however the precise role of ICS remains unclear. During the metastatic cascade, cells in primary tumor adopt mesenchymal-like features and are then able to leave the primary tumor and colonize in other locations, such as in mouse breast or pancreatic cancer models where Snail1-positive or ‘EMTing’ cells are sometimes fully detached from epithelial islands while losing E-cad expression [51]. The EMT-TF Zeb1 has been shown to be important for promoting a spectra of tumor types from mesenchymal, mixed (possibly at ICS), and epithelial as its loss leads to a confined epithelial state in pancreatic cancer [52]. *In vivo* live cell imaging coupled with gene expression analysis indicates that cells disseminated from primary tumors exhibit low expression of E-cad and enhanced expression of mesenchymal markers [53], suggesting that those cells have characteristics of ICSs. Interestingly, in a pancreatic cancer model, deletion of Twist or Snail1 does not cause any alterations in invasiveness [54], indicating cells (possibly ICS) that lose some of the well-studied mesenchymal markers may possess mesenchymal-like features. Along the same lines, in a lung metastases model, although EMT occurs within the primary epithelial tumor, the initial lung metastases are mainly derived from non-EMT tumor cells [55], and EMT cells do play a major role in recurrent lung metastasis after chemotherapy [55]. Beyond the gain of mesenchymal-like features during primary tumor dissemination, dynamic EMT gene expression is also observed in circulating breast tumor cells [29]. Together these data suggest that during EMT there are cells other than mesenchymal cells showing mesenchymal-like features with tissue and context specific roles during metastasis. Further analysis on such cells and their connection with ICS is important for better understanding of metastasis.

### 4.5. Speculated roles of ICS: controlling noise and dynamical robustness

Mathematical modeling and computational analysis have shown that ICS has the ability to control noise and increase the robustness of EMT dynamics. Increased number of intermediate phenotypes in the EMT system can better attenuate the overall fluctuations of the cell population in terms of phenotypic compositions, thereby stabilizing a heterogeneous cell population on the EMT spectrum, via a dynamic ODE modeling of the population of each cell phenotype [56]. The existence of ICS can also allow noise attenuation while maintaining the mean of the signal [57]. In another example using a regulatory circuit of miR-200, Zeb, and Snail1, the ICS is observed to increase the plasticity of cell fate and the robustness of EMT dynamics [58]. With ICS, the EMT process can be carried out through transition first from the epithelial state to a ICS temporarily, and making further transition then from the ICS to the mesenchymal state or directly going from the epithelial state to the mesenchymal state. Moreover, cells at ICS can go back to epithelial state at other times, depending on the signal inputs for flexibility [58].

### 4.6. Stability

The ICS of EMT has been considered ‘metastable’ [35, 59], reflecting the flexibility of these cells to undergo or reverse the EMT process [60]. In angiogenesis, hybrid tip/stalk phenotype, resulting from higher production levels of Jagged, relates with poorly perfused and chaotic angiogenesis based on the theoretical framework for Notch-Delta-Jagged-VEGF signaling [61]. But some evidence show that ICS could be stable. Experimentally, H1975 lung cancer cells can display a stable ICS over 2 months in culture [28]. In addition, the landscape of the free energy changes during EMT of the lung cancer cells shows a stable intermediate state [34]. Not only in EMT, ICS of immune system’s T-helper cells are also observed and are stable, having two canonical subtypes [62].

Interestingly, computational modeling that considers the mutual inhibitory loops between several miRNAs and EMT-TFs indicates that such networks are capable of generating additional stable ICSs [2, 24, 28] and have identified multiple phenotypic stability factors, such as Ovol, GRHL2, miR-145 [28] and NUMB, that can stabilize an intermediate E/M phenotype [63]. What’s more, ICSs can be stabilized due to the increase of gene expression noises [31, 64].

## 5. Rising issues and challenges

Recently, single-cell RNA sequencing (scRNA-Seq) has emerged as a powerful means to dissect the heterogeneity in normal and diseased epithelial tissues. The ability to sample the transcriptome and quantify gene expression changes at single-cell resolution has yielded unprecedented insights into the physiology of epithelial systems by aiding in the discovery of rare cell types, shedding light on the dynamics of lineage differentiation, and providing comprehensive gene expression profiles of diverse cell types [10,65]. scRNA-Seq has also begun to be employed to better understand the transition states that occur during EMT in cancer. In a recent study, scRNA-Seq revealed the presence of a partial EMT-like state in human squamous cell carcinoma (SCC) samples, implicating the existence of ICS *in vivo.* Another study utilized mouse models of SCC and mammary tumorigenesis for single-cell surface marker/RNA-seq analyses, and identified subpopulations of tumor cells corresponding to different degrees of EMT, with some displaying hybrid phenotypes that likely represent multiple distinct ICSs *in vivo* [66]. It is anticipated that single-cell approaches will also reveal EMT-related heterogeneity and enable the detection of ICSs in various normal epithelial tissues. Indeed, sequencing of 1916 single cells from eight different organs from E9.5 to E11.5 embryos led to the identification of epithelial-mesenchymal ICSs in all epithelial tissues sampled, including intestine, liver, lung, and skin [67].

In addition to characterizing ICSs, single-cell approaches have been used to suggest a role for EMT and/or ICS in development/differentiation and disease. Using an experimental pipeline that incorporates single-cell qPCR analysis, it was shown that human embryonic stem cells (hESCs) go through a stepwise differentiation process into hepatocytes by undergoing sequential EMT-MET with an obligatory ICS, implicating a potential role for EMT/ICS in hESC differentiation into a definitive endodermal fate [68]. The SCC cells of the distinct ICSs discussed above display different clonogenic and differentiation potentials, as well as distinct invasive and plastic properties, providing experimental evidence that the different transition states that arise from EMT progression have different biological functions [66].

It has become increasingly clear that ICSs during EMT are important biological entities and must be considered whenever EMT is studied. The exact roles of EMT, and particularly of the constituent ICSs in different developmental and disease contexts need to be investigated using a combination of experimental and computational approaches. It would be interesting to computationally model the overall dynamics of the cellular states in a continuum fashion to capture their relatively unstable properties rather than studying multiple statuses in between the main stable states. Developing sophisticated computational tools that facilitate the identification and characterization of various ICSs—cell states that are more transient and may be different from other major cell states—represents a major challenge as well as opportunity in the EMT field.
